# A Case of Leukocytoclastic Vasculitis Caused by* Listeria monocytogenes* Bacteremia

**DOI:** 10.1155/2016/1093453

**Published:** 2016-05-29

**Authors:** Daniel R. Bunker, Timothy Sullivan

**Affiliations:** ^1^Department of Medicine, Division of Rheumatology, Icahn School of Medicine at Mount Sinai, New York, NY 10029, USA; ^2^Department of Medicine, Division of Infectious Diseases, Icahn School of Medicine at Mount Sinai, New York, NY 10029, USA

## Abstract

*Importance.* Infections can cause leukocytoclastic vasculitis.* Observations.* We report the case of a patient with a left ventricular assist device who presented with acute kidney injury and biopsy proven leukocytoclastic vasculitis. Blood cultures grew* Listeria monocytogenes*. The patient's rash improved with treatment of the underlying* Listeria* infection.* Conclusion.* Clinicians should be aware that there are a number of broad categories of disease associated with the histologic finding of vasculitis, including infection. It is important to keep in mind the risk factors of a particular patient when formulating a differential diagnosis. This is the first reported case of* Listeria* bacteremia causing leukocytoclastic vasculitis.

## 1. Introduction

The histologic finding of vasculitis is not limited to autoimmune diseases. There are many other potential causes, including medications and infections. We report the first case of leukocytoclastic vasculitis caused by* Listeria monocytogenes* bacteremia.

## 2. Report of a Case

A 76-year-old male with end-stage ischemic cardiomyopathy treated with placement of a continuous flow left ventricular assist device (LVAD, HeartMate II, Thoratec Corporation, Pleasanton, CA, USA) was admitted to the hospital for acute kidney injury. The patient had been in his usual state of health until one week prior to admission when he developed progressive dyspnea on exertion and new gross hematuria. Review of systems was notable for slight subjective fever over the previous few days, mild abdominal cramping, nonbloody watery diarrhea, and a new nonpruritic, nonpainful, petechial rash involving the bilateral shins and left arm. He denied chest pain, orthopnea, or lower extremity swelling and had noted no LVAD alarms. Initial labs were notable for a creatinine of 4.08 mg/dL (previously 1.0 mg/dL five weeks earlier) and a urinalysis showed significant proteinuria (>300 mg/dL), numerous red blood cells, and occasional white blood cells (all new from previous studies). There was no leukocytosis or thrombocytopenia, and his chronic anemia was at baseline (hemoglobin 8.7 g/dL). Chest X-ray revealed mild bibasilar atelectasis but no pulmonary edema, consolidation, or nodules.

**Figure 1:**
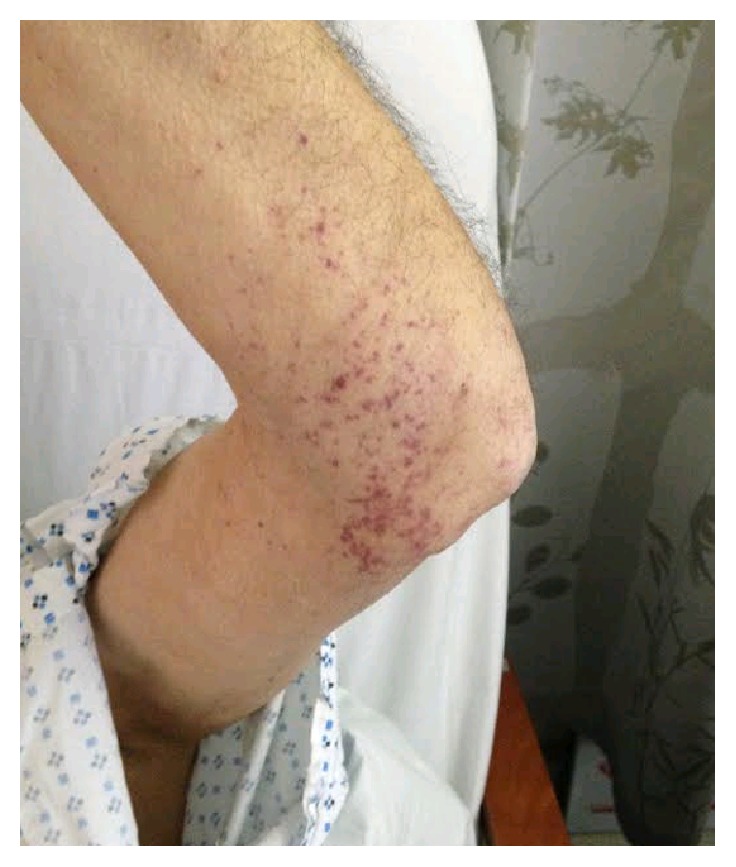
Petechial rash.

**Figure 2:**
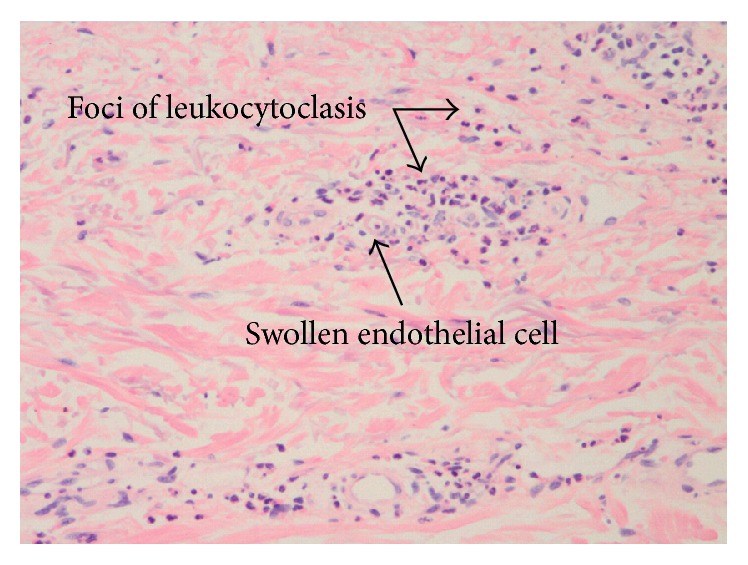
Punch biopsy skin. Punch biopsy of the skin shows swollen endothelial cells and foci of leukocytoclasis consistent with early leukocytoclastic vasculitis.

The rheumatology service was called to determine if a systemic vasculitis was causing his new acute kidney injury and petechial rash. The patient denied any history of chronic daily fevers, night sweats, or weight loss and any history of sinusitis, cough or hemoptysis, joint pains or swelling, peripheral sensory abnormalities, or focal muscle weakness. His exam showed an afebrile elderly male in no acute distress, without any hearing loss, sinus tenderness, nasal perforation, oral ulcers, pulmonary crackles, synovitis, decrease in light touch sensation, or muscle weakness. His skin was notable for nonblanching petechiae involving the left elbow (Figure 1), wrist, and bilateral shins, without mucous membrane involvement. Punch biopsy of the skin of the left wrist demonstrated early leukocytoclastic vasculitis (Figure 2).

Because of concern for an idiopathic inflammatory small vessel vasculitis causing acute glomerulonephritis, pulse dose steroid therapy was started with IV methylprednisolone 1 gm daily. However, blood cultures drawn on the day of evaluation grew* Listeria monocytogenes* after 16 hours; on further questioning, he admitted to ingestion of unpasteurized cheese at home. Anti-neutrophilic cytoplasmic antibodies, anti-nuclear antibodies, and serum cryoglobulins were normal. C3 was mildly low at 61 mg/dL, but C4 was normal. The* Listeria* bacteremia was felt to explain both his leukocytoclastic vasculitis and his acute kidney injury, so pulse steroid therapy was discontinued, while ampicillin was initiated. Though blood cultures were positive for three consecutive days, no evidence of endocarditis was seen on transesophageal echocardiogram. A renal biopsy showed acute glomerulonephritis with isolated C3 deposits, consistent with an infectious glomerulonephritis. The patient was treated with four weeks of ampicillin and cultures remained negative. His rash resolved; however, his renal function did not recover and long term hemodialysis was initiated. One month after discharge, he was readmitted for acute confusion during an episode of dialysis. Blood cultures again grew* Listeria monocytogenes* and again cleared with ampicillin. Unfortunately during that admission, he suffered a large left subdural hematoma that progressed rapidly to intraparenchymal hemorrhage and left uncal herniation, and he expired.

## 3. Discussion

Leukocytoclastic vasculitis refers to the histologic finding of neutrophilic inflammation in postcapillary venules, associated with fibrinoid necrosis, endothelial swelling, and red blood cell extravasation [1]. It is often used interchangeably with cutaneous small vessel vasculitis; the term “leukocytoclastic” describes the fragmentation of nuclei caused by apoptosis of the infiltrating granulocytes [2]. The underlying causes of this histologic finding are diverse [3]. Infections can cause a small vessel vasculitis either directly via invasion of the endothelium as in rickettsial infection or indirectly by generating immune complexes [4]. Though there have been many reports of leukocytoclastic vasculitis secondary to endocarditis [5] and/or bacteremia [6], this appears to be an overall uncommon cause: a recent single center review found that only two of 84 cases of biopsy proven leukocytoclastic vasculitis were caused by a bacterial infection (both streptococcal) [7]. To our knowledge, this is the first reported case of* Listeria* bacteremia causing a leukocytoclastic vasculitis.

Mechanical support devices for end-stage heart failure such as left ventricular assist devices (LVADs) are increasingly used both as a bridge to transplant and as destination therapy. Although these devices may be life sustaining, serious complications are common. Infections related to device placement, ranging from driveline infections to endocarditis, can affect at least one-third of patients [8], and while the risk of device related infections is lower with newer continuous flow designs, longitudinal studies still suggest that up to 15% of patients with these LVADs will develop bacteremia at some point after implantation [9]. In our patient, the repeat episode of bacteremia with the same organism suggests endovascular seeding. Indeed, glomerulonephritis with prominent C3 deposition, as seen in our patient, is a common biopsy finding in kidney injury related to endocarditis [10]. Though transesophageal echocardiogram did not show vegetation during his initial hospitalization, the reflective surface of an LVAD can impair echocardiographic visualization [11]. Although glomerular disease caused by LVAD infection may be uncommon, a report of an LVAD chronically infected with* Corynebacterium jeikeium* leading to mesangial proliferative glomerulonephritis was recently described [12]. Furthermore, the pathophysiology may be similar to that of the previously described phenomenon of “shunt nephritis,” in which a chronically infected implanted device leads to the deposition of circulating antigen-antibody immune complexes in the kidney.

*Listeria monocytogenes* is a gram-positive organism that is ubiquitous in soil and is relatively unique among foodborne pathogens in its ability to grow at the cold temperatures used in refrigeration [13]. Cheese has been the source of a number of outbreaks [14]. Though* Listeria* can cause a febrile gastroenteritis in healthy people, it may cause severe infections in pregnant women, neonates, and other immunocompromised patients, especially those with impairments in cell-mediated immunity [15]. Studies of T cell activation suggest that patients with LVADs suffer from persistent deficits in cellular immunity [16], potentially increasing their risk of infection beyond the baseline risk associated with foreign device placement. However, this deficit may be less pronounced with continuous flow devices than older pulsatile flow devices [17].

## 4. Conclusion

Clinicians should be aware that there are a number of broad categories of disease associated with the histologic finding of vasculitis, including infection. It is important to keep in mind the risk factors of a particular patient when formulating a differential diagnosis. In our patient, given his underlying LVAD placement, an infection was in retrospect the more likely cause of his vasculitis seen on skin biopsy than a de novo autoimmune disease. Though rare,* Listeria* bacteremia should be included in the differential diagnosis in patients with the histologic findings of leukocytoclastic vasculitis.
